# Breaking through the glioblastoma micro-environment via extracellular vesicles

**DOI:** 10.1038/s41388-020-1308-2

**Published:** 2020-05-04

**Authors:** Thomas Simon, Ellen Jackson, Georgios Giamas

**Affiliations:** Department of Biochemistry and Biomedicine, School of Life Sciences, University of Sussex, Brighton, BN1 9QG UK

**Keywords:** CNS cancer, Head and neck cancer

## Abstract

Glioblastoma (GBM) is the most common and most aggressive brain tumour. Prognosis remains poor, despite the combined treatment of radio- and chemotherapy following surgical removal. GBM cells coexist with normal non-neoplastic cells, including endothelial cells, astrocytes and immune cells, constituting a complex and dynamic tumour micro-environment (TME). Extracellular vesicles (EVs) provide a critical means of bidirectional inter-cellular communication in the TME. Through delivery of a diverse range of genomic, lipidomic and proteomic cargo to neighbouring and distant cells, EVs can alter the phenotype and function of the recipient cell. As such, EVs have demonstrated their role in promoting angiogenesis, immune suppression, invasion, migration, drug resistance and GBM recurrence. Moreover, EVs can reflect the phenotype of the cells within the TME. Thus, in conjunction with their accessibility in biofluids, they can potentially serve as a biomarker reservoir for patient prognosis, diagnosis and predictive therapeutic response as well as treatment follow-up. Furthermore, together with the ability of EVs to cross the blood–brain barrier undeterred and through the exploitation of their cargo, EVs may provide an effective mean of drug delivery to the target site. Unveiling the mechanisms by which EVs within the GBM TME are secreted and target recipient cells may offer an indispensable understanding of GBM that holds the potential to provide a better prognosis and overall quality of life for GBM patients.

## Introduction

Glioblastoma (GBM) is the most common, aggressive primary adult brain tumour, leaving patients with a median survival rate of 15 months after treatment, while displaying a high recurrence rate and resistance to therapeutics [1]. The current treatment for GBM patients includes maximal surgical resection followed by radiotherapy with temozolomide (TMZ), an oral alkylating chemotherapy drug [2]. Unfortunately, GBM usually recurs at or near the primary site within months of surgical removal [1]. Considering the low median survival rate for patients undergoing the current standardised treatment protocol, the development of new therapeutic procedures in overcoming GBM is urgently needed.

**Fig. 1:**
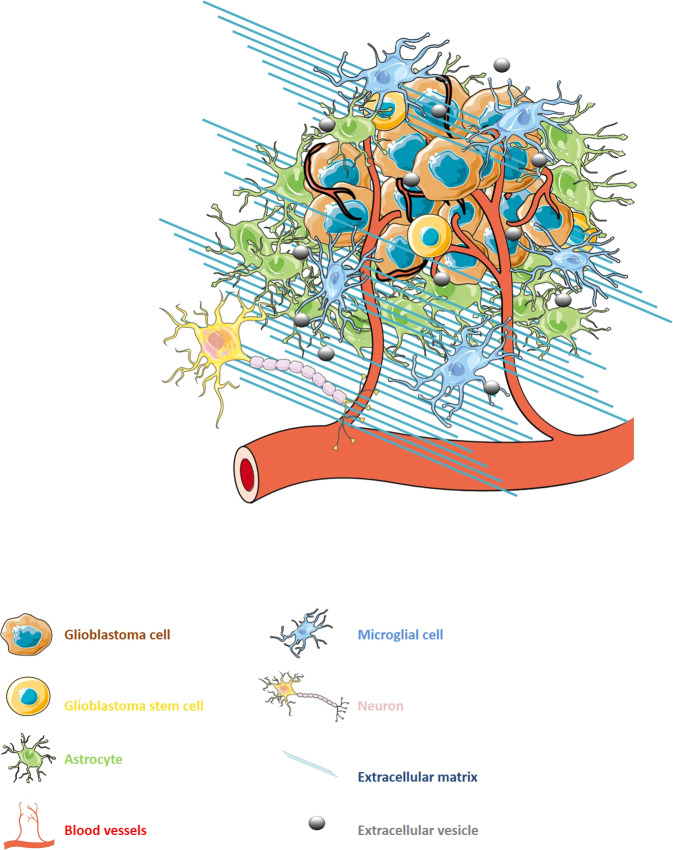
The cellular heterogeneity of the glioblastoma micro-environment. Constant multi-directional communication between glioblastoma cells and normal stromal cells such as endothelial cells or astrocytes allows the tumour to hijack its surrounding micro-environment, thus triggering a tumour supportive phenotype. Extracellular vesicles have been recently described as being directly involved in such crosstalk, along with diffusible cytokines and growth factors.

GBM exists in constant communication with its surrounding tumour micro-environment (TME) [3]. The GBM TME is highly heterogeneous, consisting of a multiplex of both cancerous and non-cancerous cells including endothelial cells (ECs), immune cells, glioma stem-like cells (GSCs) and astrocytes, as well as non-cellular components such as the extracellular matrix (ECM) (Fig. 1) [4]. The TME is becoming increasingly recognised as a crucial supporter to the progression of GBM, playing a critical role in the regulation of tumour development [5]. Extracellular vesicles (EVs) have recently been identified as an important means of bidirectional communication between the tumour and the TME [6, 7]. According to the International Society for Extracellular Vesicles, EVs are cell-released membrane-bound particles that do not contain a functional nucleus [8]. EVs encapsulate and transfer molecules including lipids, proteins and nucleic acids, amongst other bioactive materials, allowing for communication between both neighbouring and distant cells [9]. The transfer of such materials between cells in the TME, as well as tumour cells, have been shown to facilitate several tumour-promoting mechanisms including angiogenesis, invasion, evasion of apoptosis and resistance to drugs such as the aforementioned TMZ as well as bevacizumab, a human monoclonal antibody targeting vascular endothelial growth factor-A (VEGF-A) [10, 11].

GBM is typically classified into three subtypes, namely proneural, classical and mesenchymal, according to the gene expression of various biomarkers including platelet-derived growth factor receptor (PDGFR), neurofilament light chain (NF-L), epidermal growth factor receptor (EGFR) and CD44, respectively [12]. Unfortunately, the current gold standard for GBM diagnosis is highly invasive and unrepresentative of GBMs extreme intra-tumoural heterogeneity [13]. As their content seems to reflect that of the donor cell, EVs have been proposed as potential non-invasive biomarkers [12]. In addition, their ability to be detected in the bloodstream, cerebrospinal fluid and urine of GBM patients make EVs valuable candidates for a liquid biopsy set up [14]. Furthermore, EVs capability to cross the blood–brain barrier (BBB) is an important factor in considering them for the successful delivery of drugs to the TME and tumour site [15]. Altogether, such potential ability of EVs to better represent the heterogeneity of the tumour and the TME thus renders them a desirable and effective candidate for GBM subtyping and predicting therapeutic response [12].

Through influencing the TME, EVs contribute considerably to the regulation of tumour progression [16, 17]. Unveiling the impact of EVs in the TME and overall tumour progression is altogether paramount for the development of novel and effective treatment strategies against GBM. This review will thereby underpin the mechanistic interactions between GBM cells and their stromal counterparts via EVs. Furthermore, the role EVs could play in GBM therapeutic resistance will be discussed. Thereafter, the exploitation of EVs for GBM subtyping and, in turn, the construction of personalised, more effective treatments for GBM patients will be explored.

## Extracellular vesicle-based interactions of glioblastoma cells with stromal counterparts in the tumour micro-environment

### Implication of extracellular vesicles in the neo-angiogenesis process associated with glioblastoma development

GBM radical and rapid growth eventually leads to the formation of areas of extreme hypoxia [18]. Such oxygen deprivation promotes neo-angiogenesis mechanisms in the GBM TME, in order to provide oxygen and nutrients for tumour growth and survival. GBM is critically dependent upon angiogenesis and hence is one of the most highly vascularised tumours [19]. For angiogenesis to occur, ECs are required to proliferate, migrate and form tubular structures [20]. EVs derived from GBM cells have been implicated in EC proliferation, migration and tubulogenesis via delivery of angiogenic proteins and RNA to microvascular ECs [21].

**Fig. 2:**
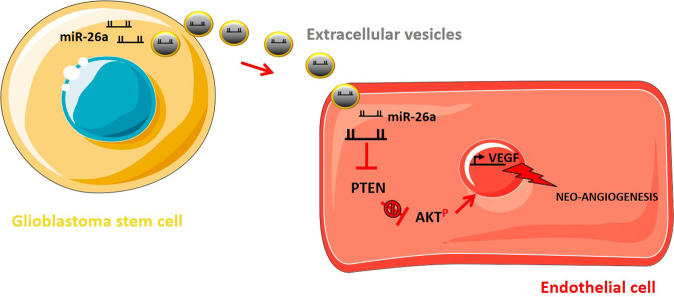
Glioblastoma stem cell-derived extracellular vesicles can stimulate neo-angiogenesis via miR-26a (Adapted from Wang et al. [25]). Extracellular vesicles derived from glioblastoma stem cells transfer miR-26a to endothelial cells, consequently inhibiting phosphatase and tensin homolog (PTEN) and promoting the activation of the PI3K/AKT pathway. Such activation upregulates the VEGF expression with consecutive promotion of the neo-angiogenesis process.

Mostly released by hypoxic GBM cells, VEGF-A promotes the proliferation and migration of ECs toward hypoxic regions of GBM [22]. It has been reported that EVs containing VEGF-A, secreted from GSCs, trigger angiogenic properties in ECs in vitro, thereby promoting and sustaining vasculature in the GBM TME [23]. Furthermore, Sun et al. observed that EVs derived from GSCs transfected with miR-21 (GSC–EV^miR-21^) promote the ability of ECs to migrate and form tubular structures. Accordingly, a significant reduction in EC tube formation ensued following the knockdown of VEGF, hence implicating VEGF in the mediation of GSC–EV^miR-21^-associated neo-angiogenesis. Furthermore, as VEGFR2 is highly accountable for the downstream angiogenic properties of VEGF, the authors also showed that the interaction of GSC–EV^miR-21^ with ECs markedly promotes the activation of VEGFR2 in ECs [24]. In the same way, Wang et al. demonstrated in vitro that miR-26a can be transferred from GSCs to ECs via EVs. It was shown in vivo that the overexpression of miR-26a in GSCs enhanced the angiogenic properties of ECs and tumour growth in nude mice, accompanied by an elevation in VEGF levels. Indeed, western blotting analysis revealed that the overexpression of miR-26a induced a reduction in PTEN, thereby activating the PI3K/AKT pathway. Consequently, it was concluded that miR-26a delivered in GSC–EVs to ECs triggers the PI3K/AKT signalling pathway in ECs through inhibition of PTEN, thereby upregulating VEGF and subsequently promoting neo-angiogenesis [25] (Fig. 2). Other growth factors, such as endothelial growth factor (EGF), fibroblast growth factor (FGF) and platelet-derived growth factor (PDGF) have also been implicated in EV-dependent GBM angiogenesis mechanisms [11, 25].

EV-based delivery of long intervening/intergenic non-coding RNA POU3F3 (lincPOU3F3) as well as long non-coding RNA CCAT2 (linc-CCAT2) to ECs has also been observed to trigger angiogenesis in the GBM TME in vitro and in vivo [26, 27]. GBM–EVs overexpressing linc-CCAT2 from the U87 GBM cell line were indeed reported to enhance EC expression of VEGF-A and transforming growth factor-beta (TGF-β) while inhibiting apoptosis via activating B cell lymphoma-2 (Bcl-2) and suppressing Bcl2-associated protein x (Bax) and caspase-3 in vitro [27]. Moreover, long non-coding RNA HOX antisense intergenic RNA (HOTAIR) was demonstrated to promote neo-angiogenesis in vitro by inducing the expression of VEGF-A in GBM cells but also by travelling via EVs from tumour cells to ECs [28]. Recently, a report, similarly, showed that the promotion of the neo-angiogenesis process by miRNAs, including miR-9, delivered via GBM–EVs was mostly dominated by the downregulation of anti-angiogenic pathways in recipient ECs [29].

In contrast, EVs containing miR-1 transferred between GBM cells and the TME have been shown to have tumour suppressive effects, hence decreasing angiogenesis, tumourigenicity and invasion in vitro and in vivo [16]. The overexpression of miR-1 in donor cells in vitro was thus demonstrated to be loaded into EVs and transported to recipient cells, then decreasing the recruitment of ECs and reducing neo-vascularisation in vivo. Accordingly, miR-1 levels were reported to be downregulated in GBM patient samples [16].

As a result of neo-vascularisation, the subsequent GBM vasculature markedly differs from that of healthy vasculature, comprising a highly dense, disorganised and leaky network of vessels [30]. The irregularity of neo-angiogenesis in GBM results in areas of extreme chronic hypoxia [31], promoting necrosis and invasion of GBM cells into healthy tissue in order to evade the adverse conditions [18]. EVs have been suggested as mediators of hypoxia-driven inter-cellular communication between cancer cells and ECs, implicating the importance of EVs in hypoxic-dependent phenotypic changes of GBM vasculature and consequential invasion. As such, Kucharzewska et al. demonstrated GBM–EVs, cultured under hypoxic conditions, to alter the phenotype of ECs in order to induce angiogenesis ex vivo and in vitro [32]. In addition, hypoxic GBM cells were shown in vitro to trigger a pro-angiogenic protease activated receptor (PAR-2)-mediated heparin-binding EGF signalling, inducing paracrine activation of ECs via EVs [33]. Finally, as EV-mRNA derived from the plasma of GBM patients has been shown to reflect the tumour hypoxic status in vitro and in vivo, it has been suggested to use EVs as a potential mean of assessing the oxygenation status of GBM [32].

Altogether, EV-dependent mechanisms seem essential to the neo-angiogenesis process, which is linked to a global and integrated re-organisation of the GBM TME. Indeed, ECs are also known to interact with astrocytes in the GBM TME, for instance at the BBB. Both cell types communicate with GBM cells through the EVs, thereby promoting BBB breakdown, a critical step in the GBM invasion process [17, 34].

### Extracellular vesicle implication in the dynamic relationship between glioblastoma cells and astrocytes

Astrocytes are the most abundant glial cells, composing around 50% of the human brain volume [35]. Consequently, GBM cell-derived EVs have been implicated in altering the phenotype of normal astrocytes in the TME to exhibit tumour-supporting capabilities [17, 36]. In addition, tumour-associated astrocyte-derived EVs have been shown to directly impact tumour cells. For instance, it was shown in vivo that EVs containing miR-19a, delivered from astrocytes to tumour cells, downregulated PTEN expression, thereby upregulating brain metastasis outgrowth [37]. Furthermore, it has been reported that a crosstalk between GBM and astrocytes via EVs is critical in the evasion of tumour cell apoptosis, thus contributing to GBM aggressiveness [36]. Using patient-derived cell lines, Oushy et al. exposed normal astrocytes to GBM–EVs, showing a consecutive generation of a growth-stimulating medium packed with growth factors (such as VEGF, EGF, FGF, hepatocyte growth factor (HGF) and colony stimulating factors (CSFs)), interleukins and chemokines, promoting the growth of GBM cells in vitro [36].

Accordingly, substantial proteome changes have been observed in normal astrocytes exposed to GBM–EVs, with evident interconnectivity with upstream regulators such as c-Myc, p53, TGF-β1 or fibronectin 1 (FN1), described before as implicated in tumour expansion [17]. Furthermore, authors observed that the mTOR pathway, with links to a senescence-associated secretory phenotype (SASP), could be directly associated with the proteome changes triggered by GBM–EVs in astrocytes [17]. Authors thus concluded that GBM cell-derived EVs might induce the acquisition of SASP in astrocytes in vitro, in order to support tumour growth.

**Fig. 3:**
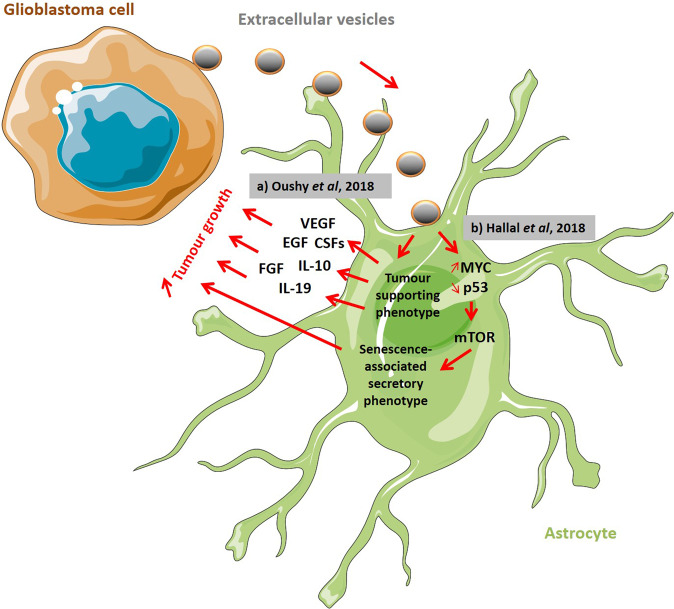
Extracellular vesicle-mediated crosstalk between glioblastoma cells and astrocytes supports tumour growth. **a** Normal astrocytes exposed to glioblastoma (GBM) cell-derived extracellular vesicles (EVs) produce a tumour growth-stimulating secretome including vascular endothelial growth factor (VEGF), epidermal growth factor (EGF), fibroblast growth factor (FGF), colony stimulating factors (CSFs), Interleukins 10 and 19 (IL-10 and IL-19) (Inspired by Oushy et al. [36]). **b** GBM cell-derived EVs influence the p53 and MYC signalling pathways in astrocytes, with consecutive acquisition of a senescence-associated secretory phenotype (SASP) in order to support tumour growth (Inspired by Hallal et al. [17]).

Furthermore, Oushy et al. observed an increase of immune response regulators, such as CSF2 and interleukin 10 (IL-10), in the secretome of GBM–EV-stimulated astrocytes, which might be indicative of a global immunosuppressive micro-environment set up, induced by GBM–EVs via tumour-associated astrocytes. Finally, both reports by Oushy et al. and Hallal et al. similarly suggested that pathways including ERK, mammalian target of rapamycin (mTOR), mitogen-activated protein kinases (MAPKs), AKT and c-Jun N-terminal kinases (JNKs) could underlie the effects of GBM–EVs on astrocytes (Fig. 3) [17, 36].

Interestingly, recent reports suggested that GBM–EVs could instigate the remodelling of astrocytic projections, thereby contributing to the invasiveness of GBM [36]. Hence, EVs from the U87 GBM cell line were shown to markedly enhance podosome formation and gelatin matrix degradation in normal astrocytes in vitro. Consequently, reports implicated these findings as a possible way via which BBB breakdown occurs in GBM patients, thereby contributing to GBM invasion [17]. Taking this into account, tumour-associated astrocytes have a promising potential to become therapeutic targets in GBM patients.

### The multiple roles of extracellular vesicles in the immunosuppressive capabilities of glioblastoma

GBM development is highly attributed to its ability to evade immune response, with GBM–EVs largely contributing to the immunosuppressive environment of GBM. GBM-derived EVs have been associated with the re-programming of microglia through the transfer of functional miR-21 in vivo. Using the GL261 mouse glioma cell line, it was shown that the mRNA targets of miR-21, such as BTG Anti-Proliferation Factor 2 (Btg2), were downregulated in microglia upon the uptake of GBM–EVs. As Btg2 is known to downregulate cyclin D1 activity, the delivery of miR-21 via EVs seems to re-program microglia into a proliferative mode. Authors suggested that such effect on microglia should influence the shaping of the GBM TME [38].

More importantly, GBM cell-derived EVs have been shown to stimulate a tumour-promoting M2 phenotype in microglia, as opposed to an immune supportive M1 phenotype [39]. GBM–EVs were therefore reported to transfer miR-451 and miR-21 to microglia cells in vitro, stimulating cell proliferation and leading to a decrease in c-Myc expression and consecutive polarisation to a M2 phenotype in the recipient cells, with increased expression of key markers such as IL-6 and tissue inhibitor of metalloproteinases-1 (TIMP-1). Through fluorescent tracking of the EVs, authors observed a similar transfer from GBM cells to microglia in vivo, with increased levels of miR-21 associated with a decrease of the c-Myc mRNA levels in brain cells [40]. Considering the wide range of genes regulated by c-Myc, authors thus concluded that such inhibition in immune cells is not an on–off switch in gene activation but is rather related with a global shift towards expression patterns associated with the activation of a tumour supportive phenotype.

More reports have demonstrated a link between EV-miRNAs and immuno-suppression in GBM. Indeed, hypoxia-induced GBM cells were reported to stimulate the differentiation of functional myeloid-derived suppressor cells (MDSCs) via delivery of EVs containing miR-29a and miR-92a both in vitro and in vivo [41]. In addition, hypoxic GBM-derived EVs containing miR-1246 were demonstrated to facilitate the formation of an immunosuppressive GBM TME through inducing M2 macrophage polarisation in targeting telomeric repeat-binding factor 2-interacting protein 1 (TERF2IP), thereby activating the signal transducer and activator of transcription 3 (STAT3) pathway and inhibiting the nuclear factor kappa B (NF-κB) pathway [42]. In the same way, GSC-derived EVs appear to promote monocytes to adopt an immunosuppressive M2 phenotype and induce the expression of programmed death-ligand 1 (PD-L1), in part mediated by STAT3. Indeed, Gabrusiewicz et al. reported that GBM patient-infiltrating CD14+ cells and monocytes treated with GSC–EVs showed increased phosphorylation of STAT3 linked to upregulation of PD-L1 expression [43]. Furthermore, GSC-derived EVs have also been implicated in mediating CD3+ T-cell suppression through inducing a monocytic MDSC/tumour-promoting phenotype in monocytes in vitro [44].

**Fig. 4:**
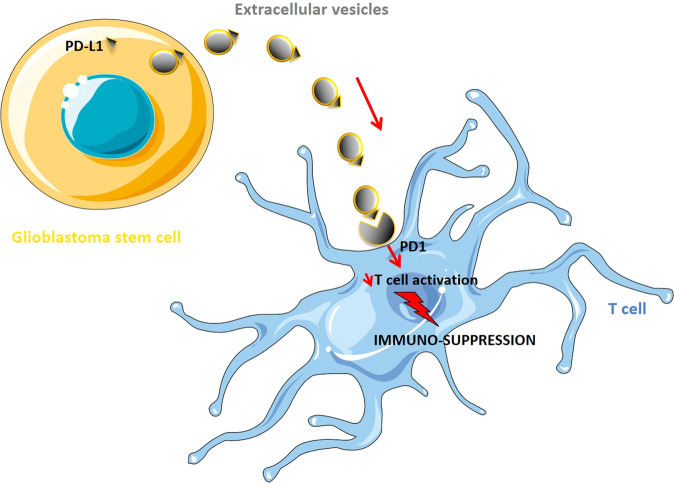
Glioblastoma cell-derived extracellular vesicles regulate the immune response to tumour growth via the PD-L1/PD1 signalling. Extracellular vesicle (EV)-associated PD-L1 can directly bind PD1 receptor at the surface of infiltrating T cells in the brain, thus inhibiting their activation and consequently supporting immunosuppression (Inspired by Ricklefs et al. [46]).

The effects of the GBM cell-derived EVs on the PD1/PD-L1 pathway also impact T-cell infiltration in the tumour and brain parenchyma [45, 46]. It has been demonstrated via in vitro and in vivo experiments that PD-L1 at the surface of GBM–EVs could prevent the activation and proliferation of T cells. Moreover, a remarkable correlation was found between PD-L1 DNA in circulating EVs and tumour volumes of up to 60 cm^3^ in GBM patient samples, suggesting the use of EV PD-L1 DNA as a biomarker for GBM. Although Ricklefs et al. conducted these experiments using GSCs, it was notably pointed out that PD-L1 is expressed by a variety of immune cells in the TME, including MDSCs, macrophages and microglia, and thus may also be significant contributors to the elevated levels of PD-L1 EVs. Conclusively, these findings demonstrate the binding of PD-L1 to PD1 on EVs to be a plausible mechanism by which GBM evades T-cell infiltration in the TME, thereby promoting GBM immuno-suppression, growth and invasion (Fig. 4) [46].

In addition, Grimaldi et al. demonstrated that microglia-derived EVs containing IFNγ could downregulate the expression of anti-inflammatory genes in microglia treated with IL-4 in vitro. Furthermore, it was shown that IFNγ–EVs could decrease the anti-inflammatory phenotype of tumour-associated myeloid cells and, consequently, significantly decreases the tumour size and neurotoxicity in the brains of mice with glioma. Such findings are suggestive of the use of microglia EVs in transference of neuroprotective signals to tumour-associated myeloid cells, thereby restoring the homoeostatic phenotype of microglia in GBM and thus homoeostasis of the CNS [47].

Overall, it appears that selective targeting of GBM–EVs involved in the conversion of M1 to M2 phenotype could thus be considered for the treatment of GBM. Mechanisms to promote the re-polarisation of an M1 phenotype in cells, possibly involving EVs, also need to be further investigated.

## Extracellular vesicles help matrix re-organisation during glioblastoma tumour progression

The ECM makes up the inter-cellular space in the CNS, comprising 20% of an adult brain and consisting of a multiplex of fibrous proteins including collagen, fibronectin and laminin, which are significant structural components of the ECM, as well as an abundance of proteoglycans and hyaluronic acid (HA) [48]. Degradation of the ECM is a critical step in the process of angiogenesis, followed by migration of ECs in forming new blood vessels [49]. ECM degradation and remodelling found in GBM patients creates a scaffold for inter-cellular communication within the TME and cell migration, triggered in response to angiogenic stimuli [50]. During GBM development ECM components such as HA and proteoglycans become overexpressed, or take on a tumour-supporting phenotype, subsequently resulting in GBM growth and invasion [19, 35, 51].

Notably, Hallal et al. found that the degradation of the ECM, which facilitates cell migration and invasion, may be induced by GBM–EVs, upregulating the formation of podosomes in normal astrocytes. Podosomes are important mediators of ECM interactions and promote ECM degradation through local proteolysis [17]. Moreover, GBM–EVs were demonstrated in vitro to contribute to the modulation of the ECM composition by decreasing p53 expression in astrocytes [17]. This loss in p53, paired with the promotion of a SASP, increases the level of FN1 [52]. Indeed, the receptor for FN1 is a integrin α5 and β1 (α5β1) dimer, known to be upregulated in GBM and associated with GBM invasion and poor prognosis [53]. Although the mechanism by which α5β1 contributes to GBM migration and invasion is not entirely known, it has been suggested to operate by means of interacting with matrix metallopeptidases (MMP) 2 [54]. Crucially, MMPs are known to be responsible for the degradation of many ECM proteins, including collagen, fibronectin and proteoglycans. Indeed, EVs have been implicated in the regulation of MMP activity [55]. For instance, EVs derived from chronically irradiated glioma cells were shown by Baulch et al. to markedly enhance MMP-2 activity in GBM cell lines. This demonstrated a possible role of EVs in the arising of a treatment-resistant subpopulation of cells after radiotherapeutic treatment which exhibited a profoundly more aggressive phenotype [56]. Furthermore, Oushy et al. suggested MMP-9 to be secreted by EV-treated astrocytes, thereby enhancing GBM invasion [36]. Also, it was demonstrated that the transfer of mir-26a in EVs from GSCs to ECs elevates MMP-2 and MMP-9 levels, thereby promoting ECM degradation and upregulation of angiogenesis [57, 58]. Furthermore, several reports on cancer cells have established CD44 to capture MMP-2 and MMP-9 at the cell surface, where they locally digest the surrounding ECM during extravasation, consequently supporting GBM aggressiveness [59, 60].

Accordingly, HA, an integral ECM component, has been strongly associated with GBM invasion and overall poor patient prognosis [51]. The binding of HA to its predominant receptor, CD44, was shown in vitro and in vivo to interact with MMPs [61], thereby driving tumour progression and upregulation of the PI3K/AKT and ERK signalling pathways, inducing pro-migratory effects and apoptotic resistance in GBM [62]. Notably, HA is more abundant in the GBM ECM than healthy brains while CD44 is often found to be upregulated in GBM tumours [60, 63]. In the same way, CD44 has been observed to be highly expressed in EVs derived from respective GBM cell lines [12]. Interestingly, the EV-mediated release of CD44 has previously been associated with the EGFRvIII mutant. Skog et al. observed EGFRvIII oncoprotein and mRNA in 47% of GBM tissue samples and found EGFRvIII in the EVs of 28% of serum samples from the same patients [64]. Further, Choi et al. reported EVs derived from parental U373 glioma cells expressing EGFRvIII to highly overexpress CD44 [65]. Thus, EGFRvIII may modulate GBM invasiveness through re-programming GBM–EVs to interact with the ECM and thereby modulate ECM components [65]. The present evidence indicates a significant role of EVs in inducing TME components to inherit a phenotype in favour of ECM modulation.

## Glioblastoma extracellular vesicles break through the blood–brain barrier

The BBB functions as a defensive interface between the brain and circulating blood, thereby allowing for the regulation of movement of cells, molecules and ions as well as limiting access of toxins and pathogens [66]. The main structure is predominantly comprised of ECs joined by tight junctions, with pericytes and astrocytes playing a crucial additional supporting role [66]. BBB is significantly altered by GBM growth, thus leading to subsequent dysfunction [67, 68]. Accordingly, GBM cell-derived EVs expressing Semaphorin3A (Sema3A) at their surface have been shown of being capable of disrupting the endothelial barrier integrity via binding to the Sema3a receptor neuropilin1 [69]. Authors observed that EVs derived from GSCs could induce re-organisation of the cell–cell adhesion molecule VE-cadherin in ECs leading to an increased permeability upon EV uptake in vitro. Interestingly, normal cell-derived EVs were not able to induce such changes. Furthermore, in vivo assays revealed that EVs derived from GBM patients’ blood carried Sema3A, consequently inducing a significant vascular leakage, as opposed to EVs derived from healthy volunteers [69]. Several other mechanisms yet to be fully described might be involved in the GBM–EVs capabilities to interact with the BBB. They might include transcytosis via which brain metastasis-derived EVs apparently can breach the intact BBB [70]. Advanced in vitro and in vivo BBB models indeed revealed that tumour cell-derived EVs are able to decrease the RAB7 levels in ECs, consecutively improving the efficacy of their own transport through the barrier via inhibiting the RAB7-driven transport to lysosomes. Interestingly, authors observed that the astrocytic component would preferentially uptake tumour cell-derived EVs promoting the BBB breach and consecutive tumour progression. Such observation thus suggested a central role of astrocytes in the BBB integrity regulation upon interactions with tumour-derived EVs [35].

Astrocytic end feet, which are indeed directly involved in the structure and tightness of the BBB, are displaced during GBM development, consequently causing loss of astrocyte-vascular coupling and formation of openings in the barrier [17, 71]. The profound change that has been observed in the astrocyte–blood vessel interactions during GBM progression, partly caused by tumour cell–EVs, might eventually have important consequences on the brain vasculature organisation, with GBM cells taking control on vascular tone [71].

Hypoxia is also a predominant driver of BBB disruption in GBM, as it directly alters the tight junctions between ECs. Indeed, the remarkable proliferation of ECs, found in GBM consecutively to increasing hypoxia, causes disruption of tight junctions, leading to the loss of BBB integrity [34]. Accordingly, VEGF-A found in hypoxic GBM–EVs has been demonstrated to increase BBB permeability in both in vitro and in vivo models. In the report published by Zhao et al., GBM–EV–VEGF-A is shown to be able to alter the expression and organisation of both claudin-5 and occludin, two of the main regulators of tight junctions, in brain microvascular ECs [34, 72]. Authors thus suggest that such disruption of the BBB via hypoxic GBM–EV could be directly involved in tumour expansion both locally and at distant sites in the brain.

Indirectly, such hypoxia-driven context has also been reported to induce changes in pericytes, which are known to be involved in the BBB structure and function. Indeed, Kucharzewska et al. observed that ECs conditioned with hypoxic GBM–EVs can stimulate pericyte migration in a paracrine way, while direct addition of GBM–EVs could not cause similar results [32]. Such data suggested that pericytes responded to a paracrine factor secreted by GBM–EV-conditioned ECs rather than to a direct stimulating signal from GBM cells. Nevertheless, EV-dependent inter-cellular crosstalk involving pericytes in the GBM micro-environment is yet to be described. However, as such interactions are better described in other cancer types where, for instance, tumour cell-derived EVs have been reported to promote the transition of pericytes into cancer-associated fibroblasts, one might expect similar mechanisms to happen in a GBM context [73].

Altogether, such alterations of the BBB structure and organisation have direct consequences on the delivery of therapeutics to the brain. The permeability of the BBB has therefore been highly studied and used in order to improve the delivery of drugs to the brain in GBM patients [15, 74]. EVs, having the ability to cross the BBB, may be considered for a safer, non-invasive drug delivery system in GBM patients. Finally, owing to their natural origin, EVs are at low risk of being immunologically rejected [4].

## Preliminary results on EV-driven interactions between tumour cells and neurons in the GBM micro-environment

Even though neurons are the most valuable and vulnerable components of the SNC, research on GBM cell–neuron interactions is still limited. Interestingly, a very elegant study reported by Gao et al. revealed that glioma cells can also interact with neurons in the TME [75]. Indeed, in vivo evidence showed that synaptic activity of neurons increased following uptake of tumour-derived EVs. Accordingly neuronal activity has been reported to be potentially involved in glioma progression, via the activity of neuroligin-3 (NLGN-3), which appears to promote tumour progression through, at least partly, the PI3K-mTOR pathway, with links to focal adhesion kinase activation [76]. Nlgn-3 knockout has thus been observed to significantly reduce tumour growth in vivo. Furthermore, authors reported that an increase of the tumour cell proliferation upon treatment with conditioned medium from optogenetically stimulated acute cortical slices was incompletely abrogated following Nlgn-3 knockout. Such results, even though they show evidence of an important role of NLGN-3 in the neuronal support to glioma growth, also suggest that other factors, potentially including EVs, might be secreted by neurons to promote tumour expansion [76]. Altogether, it thus appears that the new set of data reported by Gao et al. clearly suggest a potential involvement of EVs in a glioma cells–neurons crosstalk during glioma growth, via enhanced neuronal activity [75].

## Extracellular vesicles and glioblastoma therapeutics

### Glioblastoma extracellular vesicles as means of therapeutic resistance

**Fig. 5:**
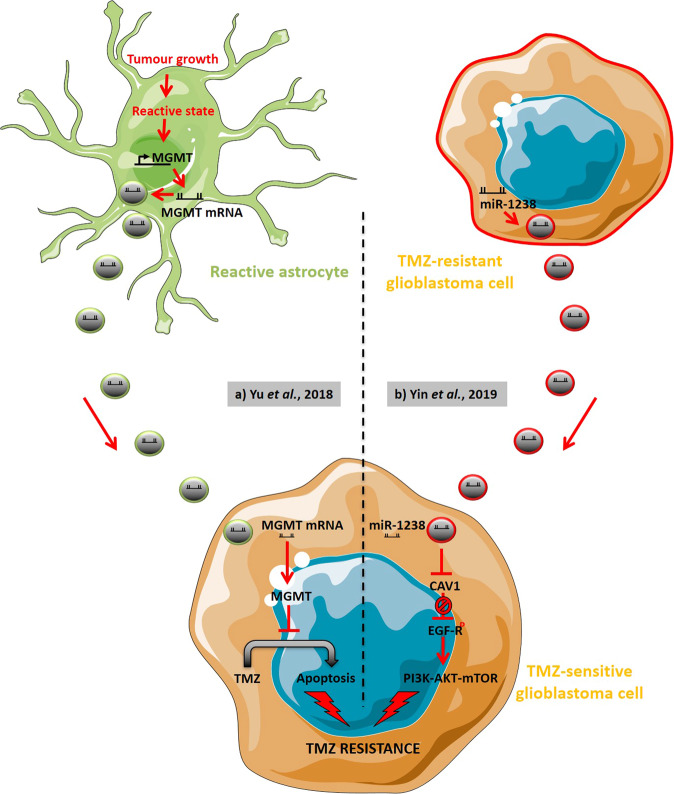
Extracellular vesicles of the glioblastoma micro-environment directly support temozolomide resistance. **a** Extracellular vesicles (EVs) derived from astrocytes in a reactive state consecutive to tumour growth can contain MGMT mRNA. Once up-taken by glioblastoma (GBM) cells, such EVs thus induce TMZ resistance via the inhibition of cell apoptosis (Adapted from Yu et al. [78]). **b** TMZ-resistant GBM cells can produce EVs carrying miR-1238 that, once up-taken by TMZ-sensitive GBM cells, inhibits caveolin-1 (CAV1), thus interfering with CAV1-driven inhibition of EGFR phosphorylation. PI3K-AKT-mTOR pathways consecutively get activated, supporting TMZ resistance (Inspired by Yin et al. [82]). TMZ Temozolomide.

The current treatments for GBM patients are of limited benefit, which can be highly accredited to GBM heterogeneity and invasiveness, as well as the ability of GBM to rapidly acquire resistance to therapies such as the alkylating agent TMZ. GBM response to TMZ is modulated by O-6-methylguanine-DNA methyltransferase (MGMT), which drives TMZ resistance when overexpressed by tumours [77]. EVs have been demonstrated to play a significant role in TMZ resistance via delivery of MGMT mRNA. Indeed, it was observed by Yu et al. that EVs derived from reactive astrocytes containing MGMT mRNA can be functionally translated into MGMT in recipient GBM cells, thereby promoting TMZ resistance both in vivo and in vitro (Fig. 5) [78].

In addition, growing evidence is unravelling the regulation of GBM chemo-resistance through EV-miRNAs [79, 80]. For instance, GBM–EV miR-93 and miR-193 have been reported to promote TMZ resistance in vitro through enhancing cell cycle quiescence by decreasing cyclin D1 [81]. In a recent study conducted by Yin et al., circulating EVs from GBM patients were found to have higher levels of miR-1238 as compared to EVs from controls. Authors reported that EV miR-1238 promoted TMZ resistance in sensitive GBM cells in vitro and in vivo, most likely involving the EGFR-PI3K-Akt-mTOR pathway (Fig. 5) [82]. In the same way, previous study conducted by Yin et al. also demonstrated the transfer of miR-151a via EVs in enhancing sensitivity of TMZ to resistant GBM cell lines through inhibiting XRCC4-mediated DNA repair [83]. Furthermore, miR-9 was also found to be upregulated in TMZ-resistant cells. Indeed, EVs of mesenchymal stem cells were shown to sensitise resistant GBM cells to TMZ through transfer of anti-miR-9, reversing the expression of multidrug transporters by reducing upregulation of miR-9. Such results actually demonstrate the possibility of utilising anti-miR delivery via EVs in therapeutics [84].

Interestingly, inhibition of EV uptake by GBM cells has been observed to be associated with reduced TMZ resistance. Indeed, as chemokine (C-C motif) receptor 8 (CCR8) was identified as an EV receptor at the surface of GBM cells, via glycans exposed on EVs and the soluble chemokine ligand C-C Motif Chemokine Ligand 8 (CCL18), inhibition of CRR8 using small molecule R243 was demonstrated to interrupt the uptake of EVs by GBM cells, thereby sensitising GBM cells to TMZ [85]. Consequently, treatment with R243 was observed to delay tumour growth in nude mice treated with TMZ. Similarly, our lab recently showed that bevacizumab, an anti-angiogenic therapeutic designed to inhibit VEGF signalling, could be encapsulated by GBM cells and presented at the surface of EVs secreted by the respective GBM cells. The inhibition of EV production was then shown to increase the effect of bevacizumab in GBM cells. We thus suggested that a combined therapeutic strategy of administering bevacizumab, together with prevention of local EV communication could enhance treatment of GBM patients [10, 19, 86].

In addition, EVs are hypothesised to be a principal driver of GBM heterogeneity. Through exchanging oncogenic cargo, EVs help alter the phenotype of various cells within the TME, including both tumour and stromal cells [87, 88]. In addition, all GBM subtypes are often present in the same tumour bulk simultaneously, thereby supporting resistance to treatments [89]. As they theoretically present the molecular background to each GBM subtype, GSCs are widely associated with GBM heterogeneity [90, 91]. For the same reasons, they have been reported as essential drivers of GBM recurrence consecutive to a mesenchymal transition of the tumour molecular background [89]. It thus has been reported that GBM–EVs may support the expansion of the mesenchymal subtype within the tumour bulk, thereby stimulating recurrence [12, 65, 87].

### Extracellular vesicles as a biomarker reservoir for glioblastoma diagnosis, prognosis and follow-up

As they are known to, at least partly, mirror the physiology of the donor cell and in turn alter the physiology of recipient cells, EVs are viewed as potential proficient indicators of changes within the TME. Accordingly, miRNA signatures and mRNA variants found in GBM–EVs have been found to be unique to GBM patients [64]. In the same way, EVs have been reported to reflect the oxygenation status of GBM cells and thus the development status of a patient’s tumour [25, 32]. Furthermore, as MGMT levels in parental cells as well as in patients seem to be mirrored by the MGMT mRNA in the respective EVs, such EV-associated MGMT mRNA could potentially serve as a real-time biomarker of chemotherapeutic response [92]. EVs have hence been put forward as reservoirs of potential biomarkers in GBM diagnosis, prognosis and treatment follow-ups, on account of their cargo specificity and accessibility in biofluids. In looking at the proteomic content of EVs, it has been suggested that GBM tumour subtyping can be established and thus may be used in directing and developing more effective and personalised GBM treatments. Our recent publication has indeed revealed that the proteomic content of EVs can mirror the molecular signature and invasiveness potential of GBM cell lines in vitro. Moreover, according to our data, EVs may contain reliable protein markers for the aggressive mesenchymal GBM subtype in particular, including CD44 [12, 93, 94]. In addition, we also observed that GBM cell-derived EVs could contain information regarding the metabolic status of the cell of origin. Accordingly, a recent report on the potential of the EV metabolic cargo as a diagnostic mean revealed interesting correlations with our own EV proteomic cargo-based sub-classification of GBM cell lines [95].

Following such idea, a protein signature has recently been identified in EVs from hypoxic U87 GBM cells in vitro, in direct association with hypoxia-inducible factor-1α (HIF-1α), transcription factor network and HIF-1 signalling [96]. The proposed hypoxic protein signature included insulin-like growth factor-binding protein 3 (IGFBP3), coagulation factor III, carbonic anhydrase 9 (CA9), glucose transporter 1 (SLC2A1), and progesterone receptor membrane component 1 (PGRMC1) mRNAs, which have previously demonstrated an involvement in GBM hypoxia and progression. As expected, this hypoxic GBM–EV signature was found to be most closely associated with the GBM mesenchymal subtype, thus suggesting that such GBM–EV hypoxic signature may be used for GBM mesenchymal subtyping [97]. Consistently, Treps et al. reported a marked increase of the VEGF-A expression in circulating EVs of GBM patients compared with those of healthy donors. Altogether, and in accordance with the EVs ability to cross the BBB, authors then argued that EV-derived VEGF-A could constitute a possible GBM diagnosis biomarker [23].

Interestingly, it appears that the expression levels of EV specific markers in the vesicles, such as CD63, CD9, heat shock protein 70 (HSP70) or annexin A2, could also be indicative of the GBM aggressiveness. We, and others, have reported that such marker expression changes are in accordance with the molecular subtype of the cells of origin. For instance, EVs produced by proneural GBM cells seem to show very low levels of CD9, CD63 and CD81 expression, as compared with EVs derived from the aggressive mesenchymal GBM cells [12, 65].

In addition, a recent study conducted by Osti et al. observed an elevated concentration of EVs in the plasma of GBM patients compared with the plasma of healthy controls. Following surgical removal of the tumour, the increased level of EVs dissipated to a concentration on par with the healthy controls. Interestingly, the increase in EV plasma concentration was resumed upon GBM recurrence [14]. Such results call attention to the diagnosis and prognosis impact of measuring EV concentration in patients’ biofluids, as a complement to describing the EV cargo.

### Use of extracellular vesicles as potential drug vehicles for glioblastoma treatment

The benefits of therapeutic drugs for GBM patients are partly impeded by the limited penetration of the BBB and poor infiltration of the GBM tumour and TME, leading to the inability to achieve effective concentrations at the site of the tumour [98]. As a result, there is a dire demand for effective drug administration to the GBM tumour site.

Several reports have thus set forth the advantages of EVs as potential drug delivery vehicles, mostly for their intrinsic abilities for bypassing the BBB [4]. In addition, as mentioned previously, their natural origin poses only low risk of immunogenic reactions and hepatic clearance [4]. Moreover, due to the lipid bilayer membrane, EVs can tolerate degrading environments and for this reason may be exploited to enhance the concentrations of drugs delivered to the target tumour site [99]. Through manipulation of their cargo, EVs could therefore be highly valuable for GBM treatment. Erkan et al. reported that EVs genetically engineered to carry the suicide gene mRNA and protein cytosine deaminase fused to uracil phosphoribosyltransferase could significantly decrease tumour expansion in mice bearing GBM tumours obtained by implantation of U87 GBM cells [100]. Finally, as some of their cargo constituents might underlie their cellular tropism, EVs could, in theory, be manipulated into highly selective drug vehicles, hence limiting side effects and toxicity on normal stromal cells in the brain micro-environment [101]. Interestingly, glycans at the vesicle surface has thus been observed to favour an uptake of EVs by CCR8-positive GBM cells [100, 102, 103]. Overall, it appears that personalised and targeted therapeutic strategies could greatly benefit from EVs in the aim to conserve healthy brain tissue typically harmed by drug toxicity during GBM treatment [101].

## Final remarks

Overall, in accordance with all the reports reviewed here, GBM–EVs seem to have the potential to unravel some of the numerous mysteries surrounding GBM high aggressiveness, complex molecular and cellular heterogeneity and discouraging resistance to therapies. But not only studying EVs can offer a glimpse into the yet to be fully described crosstalk between cancer cells and their micro-environment, it also has the potential to eventually accelerates diagnosis in a significant way via a liquid biopsy set up. Also, loading therapeutics into/onto EVs could help break on through the BBB to reach the target effectively. However, due to the extreme intra- and inter-tumoural heterogeneity of both GBM tumours and EV subpopulations, there still is a long way to go before reaching complete understanding of the complex involvement of EVs in the GBM micro-environment.

The EV field is also facing multiple limiting challenges, including reaching a consensus regarding method for vesicle concentration from biofluids and cell culture medium. Indeed, it appears that results of EV analysis are directly impacted in accordance with the methods employed to concentrate and analyse EVs [107]. Moreover, low yield and important contamination by non-EV particles dramatically impact the quality of EV samples [104]. A consensus on new methods for correcting such issues is hence urgently needed in the EV community in order to obtain bona fide and comparable research [105].

In addition, it seems critical to identify the mechanisms underlying the specific production, secretion and uptake of oncogenic and anti-tumourigenic cargo of EVs. Such understanding may hold the potential to illuminate cell–cell communication in the TME. In the same way, revealing the method of EV biogenesis and uptake may provide insight for therapeutics delivery. Finally, a therapeutic strategy to impede the secretion of EVs carrying oncogenic material may decrease invasion, immune suppression and drug resistance and thereby improve GBM patient prognosis. Again, this ultimate goal will require further extensive research into still unknown, yet essential, mechanisms such as EV cargo sorting [106].

Overall, although huge progress has been made the last two decades in understanding the roles of EVs in cancer progression in general, including extensive description of the EV cargo, there is now a need for improved and fine characterisation of the GBM–EVs, in respect of the various vesicle subpopulations and in a molecular subtype/patient-specific way. Especially in the case of the highly heterogeneous GBM tumours, associating better methods/protocols to extensive research on the vesicles biology thus seem to be the only way forward to eventually incorporate the EVs into a clinical set up for quicker and more accurate GBM diagnosis and effective, more personalised, treatments.
